# Intracholecystic papillary neoplasm associated with invasive carcinoma of the remnant gallbladder after subtotal cholecystectomy: a case report

**DOI:** 10.1186/s40792-022-01388-8

**Published:** 2022-02-21

**Authors:** Yusuke Watanabe, Naoki Mochidome, Hiromichi Nakayama, Yoshitaka Gotoh, Taro Setoguchi, Shunya Sunami, Reiko Yoneda, Yurina Ochiai, Kimihisa Mizoguchi, Hirofumi Yamamoto, Takashi Ueki

**Affiliations:** 1 Department of Surgery, Hamanomachi Hospital, 3-3-1, Nagahama, Chuo-ku, Fukuoka, 810-8539 Japan; 2 Department of Radiology, Hamanomachi Hospital, 3-3-1, Nagahama, Chuo-ku, Fukuoka, 810-8539 Japan; 3 Department of Pathology, Hamanomachi Hospital, 3-3-1, Nagahama, Chuo-ku, Fukuoka, 810-8539 Japan

**Keywords:** Intracholecystic papillary neoplasm, Intracholecystic papillary neoplasm associated with invasive carcinoma, Gallbladder cancer, Remnant gallbladder, Subtotal cholecystectomy, Remnant cholecystectomy

## Abstract

**Background:**

Intracholecystic papillary neoplasm (ICPN) of the gallbladder is a rare tumor and a relatively new concept. Therefore, the natural history and imaging characteristics of ICPN have not yet been fully documented. Moreover, cases who underwent curative resection for remnant gallbladder cancer, including ICPN with associated invasive carcinoma, have been rarely reported. We report a resected case of ICPN of the remnant gallbladder with associated invasive carcinoma for which we could observe a temporal change in imaging findings until malignant transformation.

**Case presentation:**

A 79-year-old female patient with a surgical history of subtotal cholecystectomy for acute cholecystitis was an ambulatory patient of our institution because of postoperative surveillance for colon cancer. Ultrasonography and computed tomography incidentally detected a small nodule in the cystic remnant gallbladder. The nodule had increased in size 3 months later; thus, additional investigations were performed. Magnetic resonance imaging revealed a 10-mm enhanced nodule without evidence of extraluminal invasion. Diffusion-weighted magnetic resonance imaging revealed restricted diffusion of the lesion, and positron emission tomography revealed marked accumulation in the lesion. The lesion was diagnosed as suspicious for a malignant remnant gallbladder tumor. Therefore, remnant cholecystectomy with gallbladder bed resection was performed. Because preoperative endoscopic retrograde cholangiography revealed a relatively long intact cystic duct, extrahepatic bile duct resection was planned to be omitted. Intraoperatively, the hepatic and duodenal side bile duct where the cystic duct diverged was taped. Using these tapes, which permitted pulling the bile duct, the cystic duct located behind the bile duct could be safely exposed. The lesion was pathologically diagnosed as biliary morphologic ICPN with associated invasive carcinoma.

**Conclusions:**

Because remnant cholecystectomy is an uncommon procedure and technically difficult, accurate preoperative investigation and surgical planning are important to prevent bile duct injury and omit extrahepatic bile duct resection. In the present case, intracystic change could be detected incidentally at an early stage because of previous remnant gallbladder producing (reconstituting) subtotal cholecystectomy and surveillance for other disease. This case suggests the existence of ICPN that can progress to invasive carcinoma during a short period.

## Background

Intracholecystic papillary neoplasm (ICPN) of the gallbladder is a neoplastic lesion characterized by papillary growth in the gallbladder [1, 2]. ICPN is defined as a gallbladder lesion of an intraductal papillary neoplasm of the bile duct and is a relatively new concept. Because of its rarity, the natural history and imaging characteristics of ICPN have not yet been fully documented.

When performing surgery for severe cholecystitis, subtotal cholecystectomy is an established bail-out procedure to avoid bile duct injury [3]. Few patients who undergo subtotal cholecystectomy require reoperation, including remnant cholecystectomy, which is an uncommon procedure [4]. The major reasons for reoperation for a remnant gallbladder are related to retained or recurrent biliary stones, and curative remnant cholecystectomy for remnant gallbladder cancer has been rarely reported [4–6].

Herein, we report a case of ICPN with associated invasive carcinoma of the remnant gallbladder after subtotal cholecystectomy for acute cholecystitis in which we could observe a temporal change in imaging findings until malignant transformation.

## Case presentation

A 79-year-old woman was an ambulatory patient of our institution because of postoperative surveillance for thyroid cancer and sigmoid colon cancer. She had a medical history of hypertension, chronic hepatitis C, and Parkinson’s disease, and a surgical history of subtotal cholecystectomy for acute cholecystitis at 69 years of age (this surgery was performed at another institution), total thyroidectomy for thyroid cancer at 69 years of age, and laparoscopic sigmoidectomy for sigmoid colon cancer at 78 years of age. The patient had no other comorbidities, such as diabetes, and she did not have a history of drinking alcohol and smoking. In regard to her activities of daily living, she walked with a cane and traveled middle to long distances using a wheelchair because of Parkinson’s disease. Abdominal ultrasonography (AUS) and contrast-enhanced computed tomography (CECT) were performed 6 months after the sigmoid colon surgery. Images revealed a small nodule in the cystic remnant gallbladder, which was a suspected gallstone or polyp. However, AUS and CECT performed 9 months after the colon surgery revealed an increase in the size of the nodule.

**Fig. 1:**
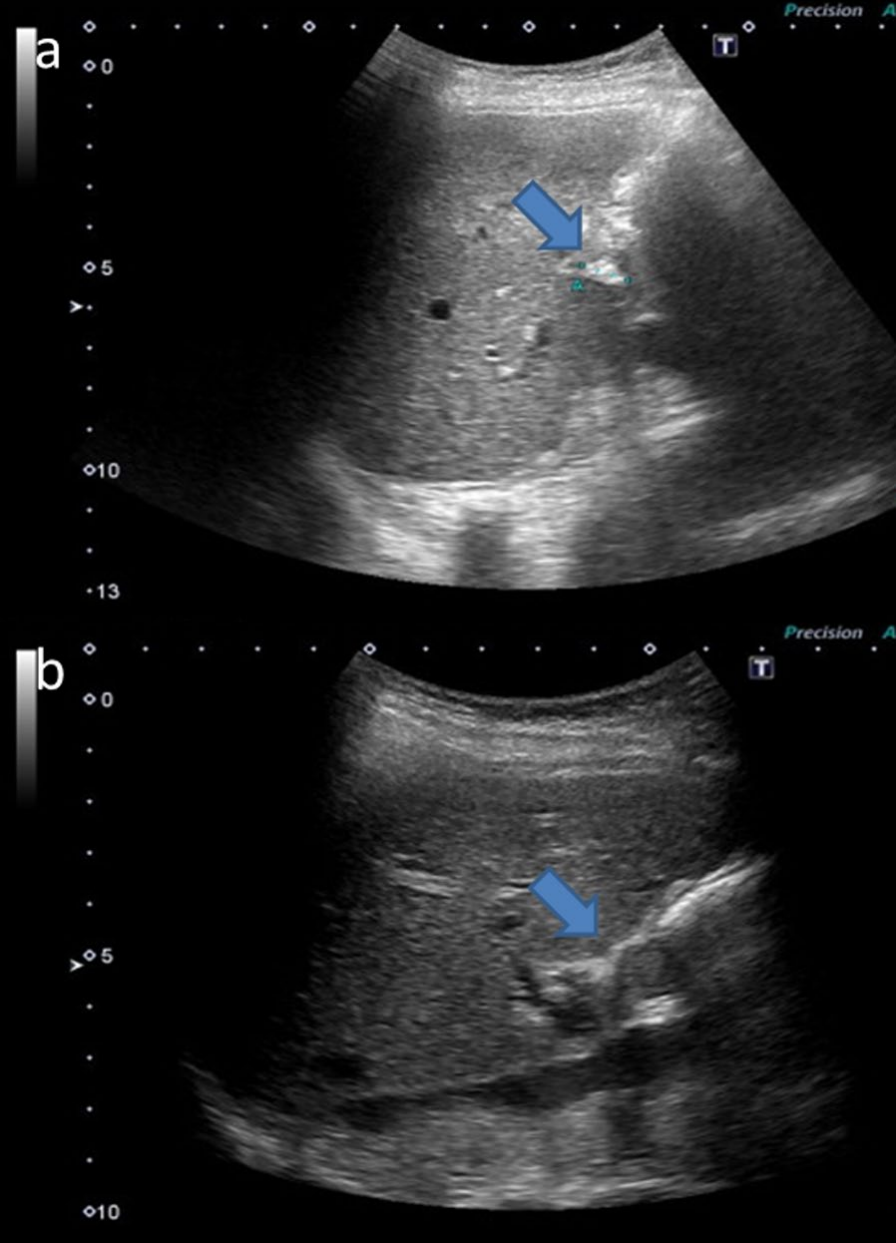
Abdominal ultrasound (AUS) findings. **a** AUS performed 6 months after the colon surgery. A Highly echoic structure is visible in the cystic remnant gallbladder (arrow). **b** AUS performed 9 months after the colon surgery. A 13-mm isoechoic solid lesion is visible in the remnant gallbladder (arrow)

**Fig. 2:**
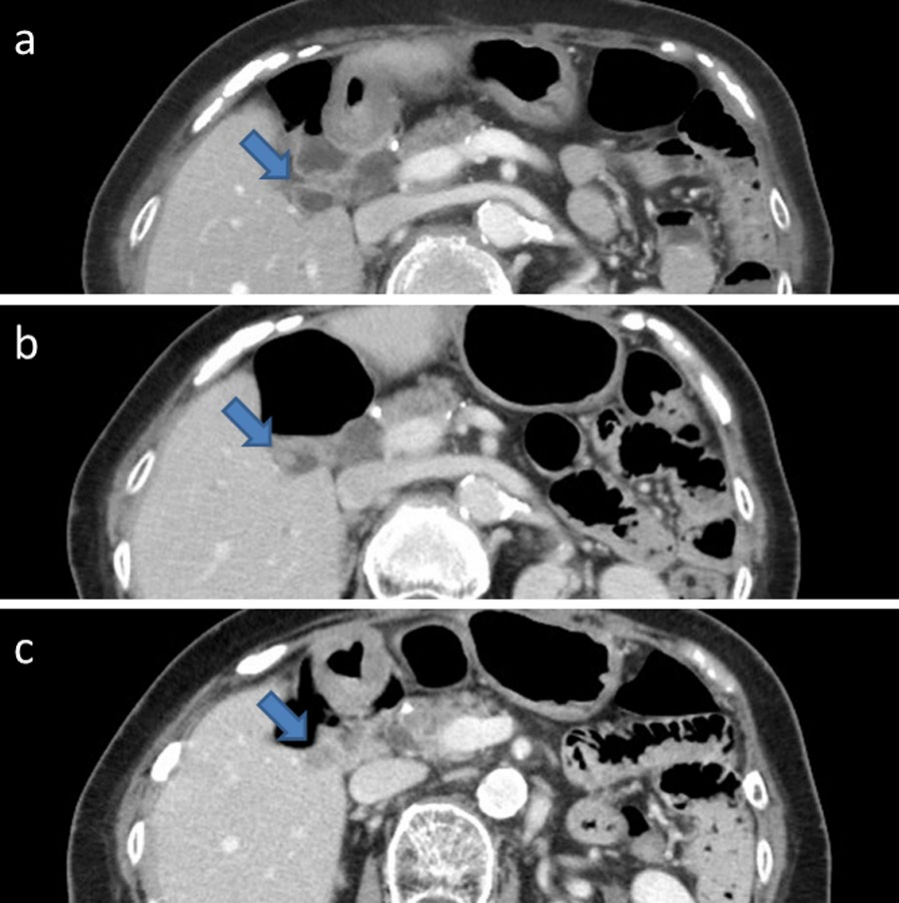
Contrast-enhanced computed tomography (CECT) findings. **a** Preoperative CECT for sigmoid colon cancer. The cystic remnant gallbladder without a nodule is visible (arrow). **b** CECT performed 6 months after the colon surgery. A 5-mm small nodule is visible in the remnant gallbladder (arrow). **c** CECT performed 9 months after the colon surgery. A 10-mm enhanced nodule is visible in the remnant gallbladder (arrow)

AUS performed 6 months after the colon surgery revealed a highly echoic structure in the cystic remnant gallbladder that was a suspected gallstone (Fig. 1a). AUS performed 9 months after the colon surgery revealed a 13-mm isoechoic solid lesion in the remnant gallbladder (Fig. 1b). Preoperative CECT for sigmoid colon cancer revealed a cystic remnant gallbladder without a nodule (Fig. 2a); however, CECT performed 6 months after the colon surgery revealed a 5-mm nodule in the remnant gallbladder that was a suspected gallstone or polyp (Fig. 2b). CECT performed 9 months after the colon surgery revealed a 10-mm enhanced nodule in the remnant gallbladder (Fig. 2c). According to these temporal changes in the AUS and CECT findings, the lesion was a suspected remnant gallbladder tumor. Therefore, additional investigations were performed.

**Fig. 3:**
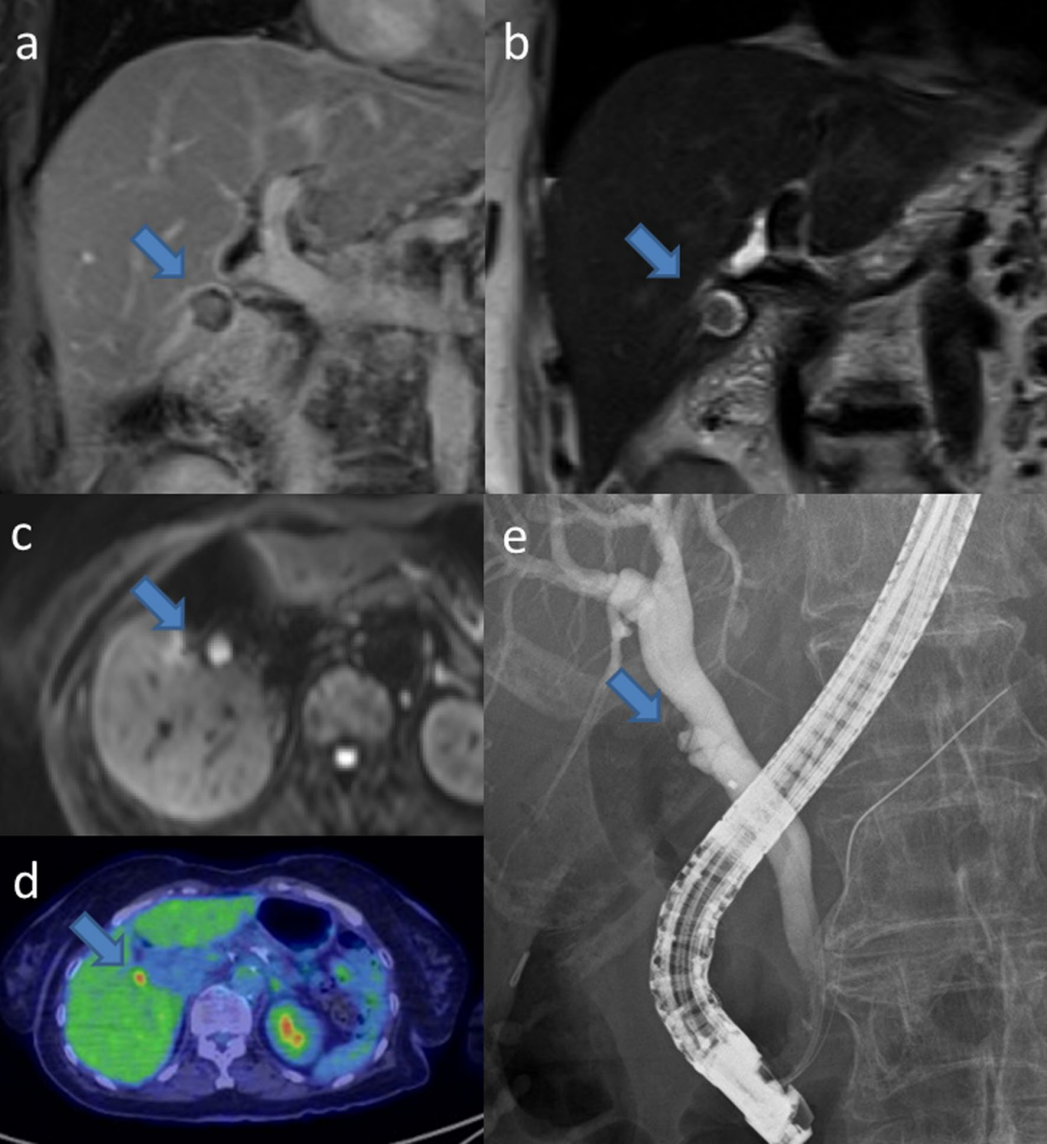
Findings of additional imaging studies. **a** T1-weighted magnetic resonance imaging (MRI). A 10-mm enhanced nodule is visible (arrow) without evidence of extramural invasion. **b** T2-weighted MRI. The lesion is detected as a filling defect (arrow). **c** Diffusion-weighted MRI. Restricted diffusion of the lesion is observed (arrow). **d** 18-fluoro-2-deoxy-glucose positron emission tomography/computed tomography (FDG-PET/CT). FDG accumulation (maximum standardized uptake value: 6.90) in the remnant gallbladder lesion is observed (arrow). **e** Endoscopic retrograde cholangiopancreatography. The cystic duct, which diverged from the left side of the middle bile duct, was confirmed. Findings suspicious for invasion to the bile duct and pancreatobiliary maljunction are not observed. Contrast medium did not flow into the remnant gallbladder via the cystic duct (arrow). The length of the intact cystic duct was approximately 15 mm. According to the cholangiography findings, extrahepatic bile duct resection was planned to be omitted

**Fig. 4:**
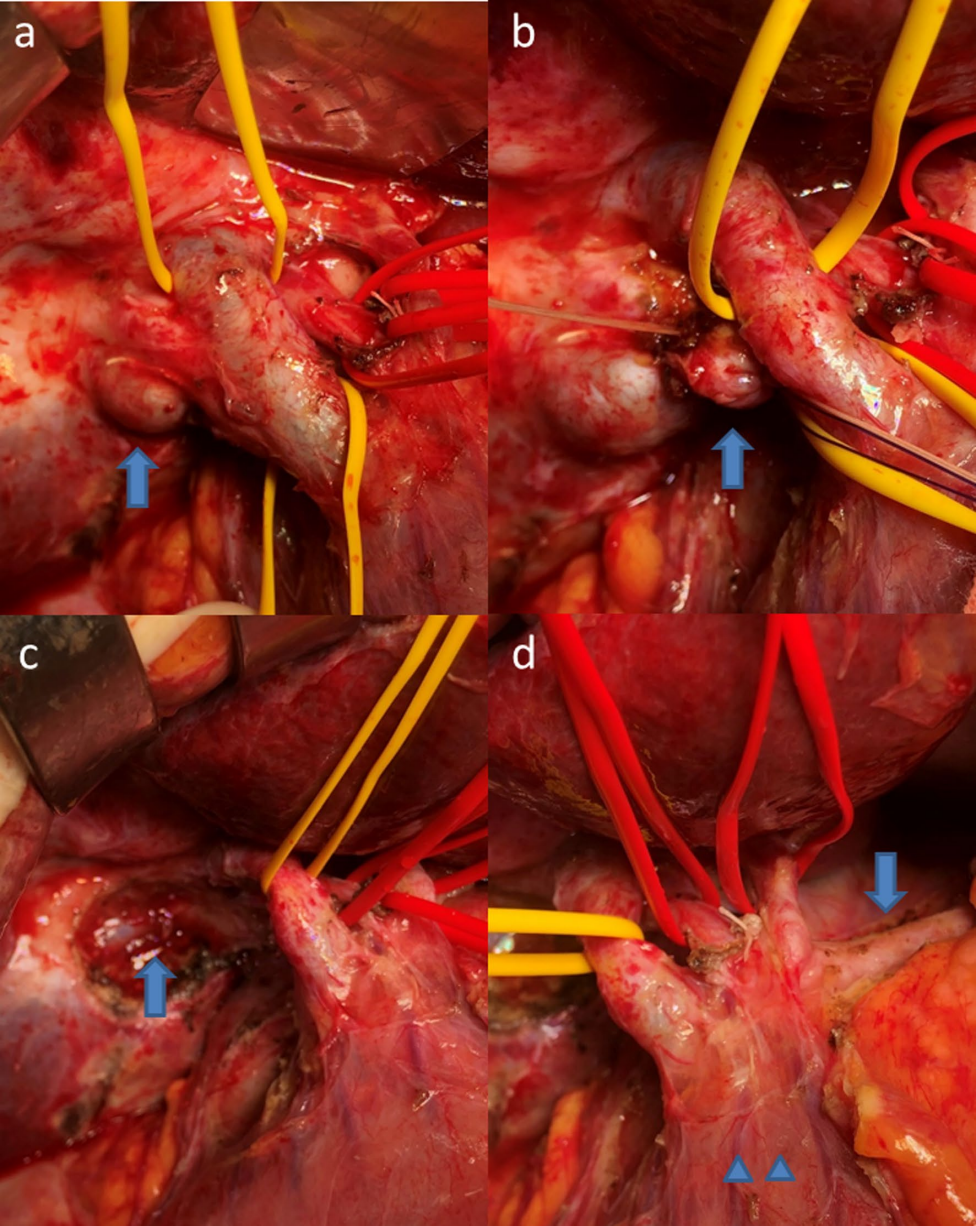
Intraoperative findings. **a** A soft mass without evidence of extramural invasion was palpable at the remnant gallbladder (arrow). During lymph node dissection around the hepatic portal region, hepatic and duodenal side bile duct where the cystic duct diverged was taped (yellow tapes). **b** Using these tapes, approximately 20 mm of the cystic duct located behind the bile duct could be safely exposed. The cystic duct was ligated (arrow) and cut, and the cystic ductal margin was negative on frozen section. **c** Remnant cholecystectomy with remnant gallbladder bed resection was performed. The right branch of the portal vein was exposed (arrow). **d** Postoperative status after remnant cholecystectomy, remnant gallbladder bed resection, and regional lymph node dissection. The arrow indicates the common hepatic artery. The right and left hepatic arteries were taped (red tapes). The upper duodenal arteries and the right gastric artery were preserved (arrowheads)

The patient had no subjective symptoms. Laboratory data indicated a decreased hemoglobin concentration of 10.3 g/dL. The remaining laboratory data, including tumor marker concentrations, were within normal limits. Magnetic resonance imaging (MRI) revealed a 10-mm enhanced nodule without evidence of extramural invasion (Fig. 3a, b). Diffusion-weighted MRI revealed restricted diffusion of the lesion (Fig. 3c), and 18-fluoro-2-deoxy-glucose positron emission tomography/computed tomography (FDG-PET/CT) revealed FDG accumulation in the lesion (Fig. 3d). Endoscopic retrograde cholangiopancreatography revealed the cystic duct diverging from the left side of the middle bile duct (Fig. 3e). No findings suspicious for invasion to the bile duct and pancreatobiliary maljunction were observed. Contrast medium did not flow into the remnant gallbladder via the cystic duct. The length of intact cystic duct was approximately 15 mm. Bile cytology did not reveal malignant cells. According to these findings, the lesion was diagnosed as suspicious for remnant gallbladder cancer. Therefore, we performed remnant cholecystectomy, remnant gallbladder bed resection, and regional lymph node dissection (Fig. 4).

An upper abdominal midline incision was made. Upper intraabdominal severe adhesions because of the previous surgery for acute cholecystitis were observed, and adhesiolysis was performed. A soft mass without evidence of extramural invasion was palpable at the remnant gallbladder. During lymph node dissection around the hepatic portal region, the hepatic and duodenal side bile duct where the cystic duct diverged was taped. Using these tapes, approximately 20 mm of the cystic duct located behind the bile duct could be safely exposed. The cystic duct was ligated and cut, and the cystic ductal margin was negative on frozen section; therefore, extrahepatic bile duct resection was omitted. Next, remnant cholecystectomy with remnant gallbladder bed resection was performed. Frozen section revealed negative surgical margins. The surgical duration was 202 min and blood loss was 35 g.

**Fig. 5:**
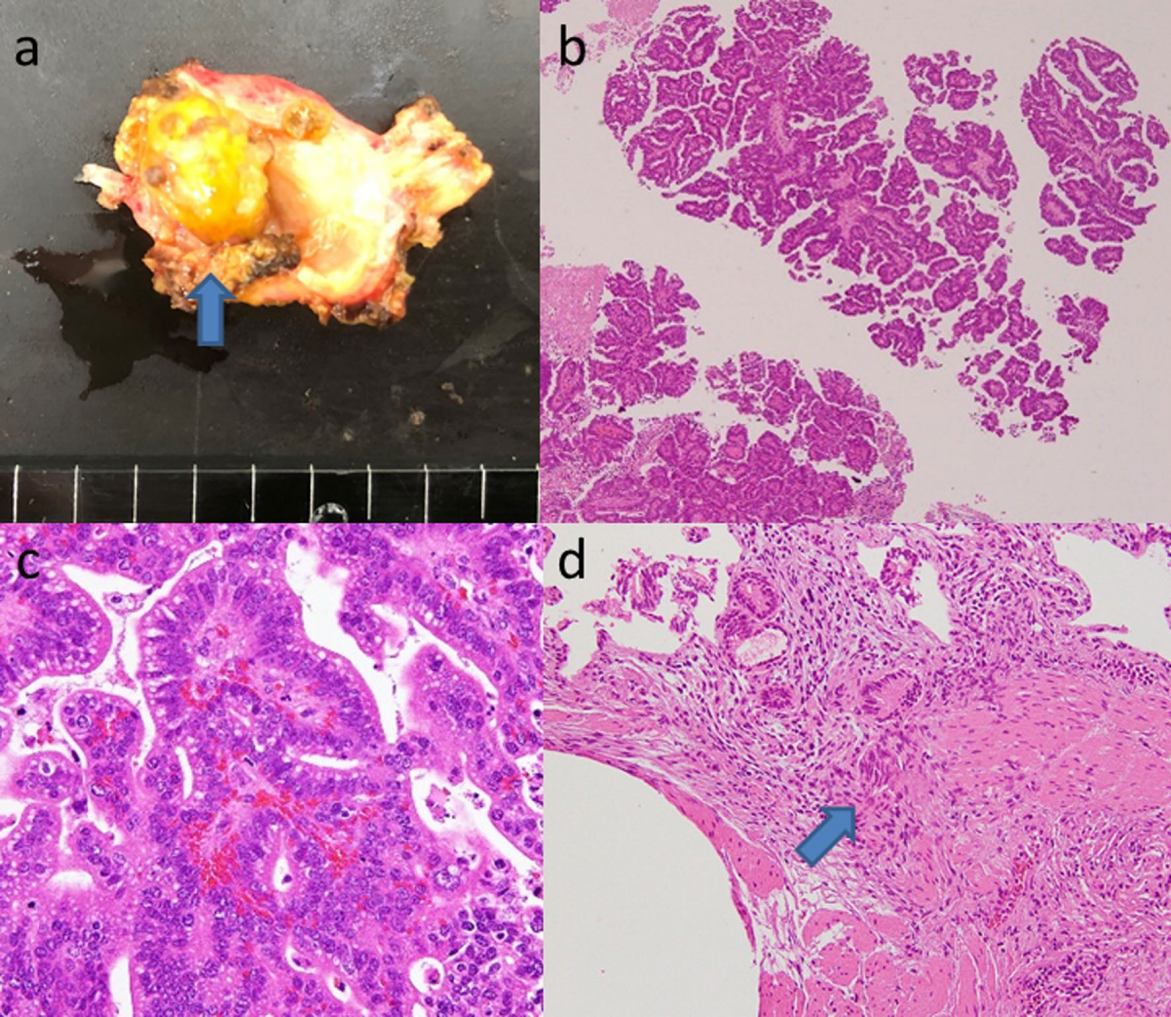
Macroscopic and microscopic findings of the resected specimen. **a** A 12-mm yellowish papillary exophytic mass (arrow) distinct from the adjacent mucosa is observed in the remnant gallbladder (the remnant gallbladder is on the left side, and the cystic duct is on the right side). **b** The tumor consisted of atypical glandular epithelium with mild-to-severe dysplasia arranged in a high papillary architecture with thin fibrovascular stalks (hematoxylin–eosin (H&E) staining, × 40). **c** Biliary morphology with cuboidal cells showing clear to eosinophilic cytoplasm, enlarged nuclei, and prominent nucleoli (H&E, × 200). **d** Focal stromal invasion in the muscle is visible (arrow). (H&E, × 200)

The macroscopic findings of the resected specimen revealed a 12-mm yellowish papillary exophytic mass distinct from the adjacent gallbladder mucosa (Fig. 5a). Microscopically, the tumor consisted of atypical glandular epithelium with mild-to-severe dysplasia with cells arranged in a highly papillary architecture along with fibrovascular stalks (Fig. 5b). Most tumor cells had biliary morphological features with cuboidal cells showing clear to eosinophilic cytoplasm, enlarged nuclei, and prominent nucleoli (Fig. 5c). Although the tumor was mostly non-invasive, focal stromal invasion in the muscle layer was observed. Thus, the lesion was diagnosed as ICPN with associated invasive carcinoma. Lymph node metastases were not observed.

Postoperatively, the patient recovered uneventfully and was discharged on postoperative day 10. At the time of this report, 8 months have passed, and the patient has experienced no recurrence.

## Discussion

ICPN is defined as a grossly visible, mass-forming, non-invasive epithelial neoplasm arising in the mucosa and projecting into the lumen of the gallbladder [1]. ICPN is defined as gallbladder lesions of intraductal papillary neoplasms of the bile duct, which is a premalignant lesion of the biliary tract and a counterpart of intraductal papillary mucinous neoplasm of the pancreas (IPMN). ICPN is more common in women older than 60 years of age and is found in 0.4% of cholecystectomies [2]. ICPN shows various degree of dysplasia from low- to high-grade and finally to invasive carcinoma, and histological findings are often mixed; therefore, this variable dysplastic degree demonstrates the adenoma–carcinoma sequence [1, 7, 8]. If there is a component of invasive carcinoma, the lesion is called ICPN with associated invasive carcinoma [1]. ICPN is classified as four morphological subtypes, namely biliary, gastric, intestinal, and oncocytic morphologies, and is separate from pyloric grand adenoma [1]. Although ICPN more commonly displays morphological heterogeneity compared with IPMN, and clinical significance of these morphological subtypes is unclear, the biliary morphology is reportedly the most common subtype [1, 2, 8]. ICPN with associated invasive carcinoma is identified in approximately half of all resected ICPNs, particularly in lesions with a predominantly biliary morphology or extensive high-grade dysplasia [2, 8]. In the present patient, the lesion was diagnosed as biliary morphologic ICPN with associated invasive carcinoma according to the predominant morphological pattern. Although ICPN is considered a precancerous lesion [9], the natural history of ICPN has not been well investigated. In this case, the accurate time when ICPN developed in the patient’s remnant gallbladder and when the lesion became invasive are uncertain. Moreover, the lesion might already have been invasive at the time of the detection of the small nodule in the remnant gallbladder. However, the present case suggests the existence of biliary morphologic ICPN that can progress to invasive disease during a short period.

ICPN without invasive carcinoma has a good prognosis after cholecystectomy. The 5-year survival rate for patients with non-invasive ICPN is 78%, whereas patients with invasive carcinoma have a 5-year survival rate of 60% [2]. Even when only ICPN with associated invasive carcinoma is considered, the overall survival outcome of ICPN is incomparably better than that of the non-ICPN-associated ordinary-type gallbladder adenocarcinoma, which has a 5-year survival rate ranging from 18 to 30% [2, 9]. In contrast, the fact that some patients with non-invasive ICPN die of biliary tract cancer, typically long after the diagnosis of ICPN, suggests that a field effect rendering the remainder of the biliary tract at risk of carcinoma [1]. This feature of ICPN resembles that of IPMN, which sometimes occurs concomitantly with pancreatic ductal adenocarcinoma [10]. Therefore, long-term surveillance is needed after resection of ICPN as well as IPMN.

Because of the rarity of ICPN, its imaging features have not been well described. Several case reports have described the imaging findings of ICPN [11–15]; however, no report has summarized the findings. According to previous reports, ICPN manifests as a polypoid lesion by AUS, and strong enhancement is observed in the early phase of contrast-enhanced studies. T2-weighted MRI of ICPN lesions reveal a filling defect, and a hypointense stalk is sometimes identified. Thickening or deformity of the gallbladder wall is rarely observed. Diffusion-weighted MRI usually shows restricted diffusion. The imaging features of FDG-PET/CT are unknown although FDG accumulation was confirmed in the present patient. The sensitivity and specificity of a ≥ 5-mm enhanced mural nodule for predicting invasive carcinoma derived from IPMN is reportedly high [16]; however, the specific preoperative imaging findings predicting ICPN with associated invasive carcinoma are unknown. ICPN is most commonly detected incidentally by imaging studies [2]; therefore, the natural imaging changes in ICPN are also unclear. The novelty of the present case is that a temporal change in the imaging findings until ICPN became invasive disease could be observed.

Cholecystectomy, including laparoscopic procedure, is difficult to perform in some patients with acute cholecystitis with severe inflammation and fibrosis [3]. The occurrence of bile duct injury and vasculobiliary injury, which affect patients’ prognosis [17], is alarming in these cases. The 2018 revised international guidelines for the management of acute cholecystitis (Tokyo Guidelines 2018) recommend subtotal cholecystectomy as a bail-out procedure to prevent iatrogenic complications [3]. Meta-analyses revealed that the rates of bile duct injury, postoperative complications, reoperation, and mortality after subtotal cholecystectomy for difficult gallbladders were low, although the rate of bile leakage was relatively high, ranging from 10.6 to 18.0% [18, 19]. Techniques for subtotal cholecystectomy have been classified as “reconstituting” when a closed remnant gallbladder is left or “fenestrating” when the remnant is left open or the internal opening of the cystic duct is closed [20]. The distinction between these procedures is whether a remnant gallbladder is produced (reconstituting) vs not produced (fenestrating). Both techniques are associated with specific complications. Bile leakage is significantly more common after fenestrating techniques, whereas the rate of recurrent biliary events is lower after fenestrating than after reconstituting techniques [21]. Additionally, the choice of a reconstituting or fenestrating procedure depends on the intraoperative conditions. The present patient previously underwent a reconstituting procedure at another institution. Because a produced cystic remnant gallbladder was left in the present patient, intracystic change might have been noticed early.

Remnant cholecystectomy is an uncommon procedure, although several researchers have reported studies of this procedure [4–6]. In these reports, the major operative indications for the remnant gallbladder related to retained or recurrent biliary stones. Although gallbladder cancer, including ICPN, can arise in the remnant gallbladder, to the best of our knowledge, this is the first resected case of ICPN associated with invasive carcinoma of the remnant gallbladder. The speculative reasons why resectable cases of remnant gallbladder cancer are extremely rare are as follows: (1) long-term surveillance after subtotal cholecystectomy is not generally performed, and in most patients who underwent subtotal cholecystectomy, clinicians were not aware of the remnant gallbladder [6]. (2) The remnant gallbladder is anatomically adjacent to major vessels. Therefore, most remnant gallbladder cancers are likely to be unresectable when patients complain of symptoms. Although long-term surveillance for all patients after subtotal cholecystectomy is not realistic, resectable invasive cancer of the remnant gallbladder could have been detected incidentally in the present case because of surveillance for other diseases.

Remnant cholecystectomy is technically difficult because of adhesions, fibrosis, and anatomical change owing to the initial surgery [4–6]. In the present case, preoperative endoscopic retrograde cholangiopancreatography was useful to evaluate the status of the cystic duct, and extrahepatic bile duct resection was planned to be omitted. Moreover, intraoperatively, the cystic duct located behind the bile duct could be safely exposed using tapes, which allowed for pulling the bile duct. Therefore, bile duct injury could be prevented and extrahepatic bile duct resection could be omitted. Accurate preoperative investigation and surgical planning are essential in similar cases.

Extrahepatic bile duct resection is performed as part of radical cholecystectomy for gallbladder cancer. However, the indication for extrahepatic bile duct resection remains a major controversy in the surgical management of gallbladder cancer that has not invaded the hepatoduodenal ligament. The 2019 clinical practice guidelines for the management of biliary tract cancers advocated by the Japanese Society of Hepato-Biliary-Pancreatic Surgery recommend not to perform routine prophylactic extrahepatic bile duct resection for gallbladder cancer without bile duct invasion [22]. Because of the patient’s age and activities of daily living, we considered that minimally invasive surgery was desirable. Moreover, preoperative imaging studies did not reveal evidence of extramural, cystic ductal, and bile ductal invasion, or evidence of regional lymph node metastasis. Therefore, extrahepatic bile duct resection was planned to be omitted in this case, although lymph node dissection around the hepatic portal region without extrahepatic bile duct resection may carry a risk of bile duct ischemia and can cause acute or chronic bile duct stenosis [23, 24]. Surgery with strict attention to conservation of blood flow to the bile duct was performed, then remnant cholecystectomy without extrahepatic bile duct resection was completed after confirmation of negative cystic ductal and surgical margins on intraoperative frozen section.

## Conclusions

In the present patient, intracystic change might have been noticed early because of previous reconstituting subtotal cholecystectomy and surveillance for other diseases. As a result, ICPN of the remnant gallbladder with associated invasive carcinoma could be curatively resected. Although the natural history of ICPN, which is considered a precancerous lesion, is still unknown, this case suggests the existence of ICPN that can progress to invasive disease during a short period.

## Data Availability

Not applicable.

## Abbreviations

ICPN: Intracholecystic papillary neoplasm
AUS: Abdominal ultrasonography
CECT: Contrast-enhanced computed tomography
MRI: Magnetic resonance imaging
FDG-PET/CT: 18-Fluoro-2-deoxy-glucose positron emission tomography/computed tomography
IPMN: Intraductal papillary mucinous neoplasm of the pancreas
